# A pharmacometric framework for norepinephrine transporter occupancy and dose equivalence across psychotropic medications

**DOI:** 10.1177/02698811261470450

**Published:** 2026-08-04

**Authors:** Mohammed Aboukaoud, Bosmat Hoch, Revital Amiaz

**Affiliations:** 1 Drora and Pinchas Zachai Division of Psychiatry, Sheba Medical Center, Ramat-Gan, Israel; 2 Gray Faculty of Medical and Health Sciences, Tel Aviv University, Ramat Aviv, Israel

**Keywords:** norepinephrine transporter, occupancy, pharmacometrics, dose equivalence, psychotropic medications

## Abstract

**Background:**

Norepinephrine transporter (NET) inhibition is a relevant mechanism across psychotropic medications, although prescribing classifications are largely based on drug class rather than quantitative target engagement.

**Aims:**

To facilitate cross-drug comparisons of noradrenergic activity, we developed a pharmacometric model to estimate NET occupancy for 26 psychotropic agents and their active metabolites.

**Methods:**

NET occupancy was estimated using National Institute of Mental Health Psychoactive Drug Screening Program Ki data, protein-binding-corrected plasma concentrations, a standard receptor occupancy model, and logit-derived ED50 values, and was compared with published positron emission tomography (PET) estimates.

**Results:**

After protein-binding correction, desipramine, milnacipran, and maprotiline showed very high NET occupancy (⩾90%), nortriptyline, doxepin, and norquetiapine showed high occupancy (70%–90%), and hydroxybupropion and duloxetine showed moderate occupancy (50%–70%). Clomipramine, atomoxetine, and reboxetine demonstrated moderate-low occupancy (30%–50%), whereas venlafaxine showed low occupancy (~28%). Hydroxybupropion exhibited substantially greater NET engagement (~68%) than bupropion (~2%), and most Selective serotonin reuptake inhibitors showed minimal occupancy (<10%). Estimated ED50 values ranged from 6.6 mg (desipramine) to ⩾63 mg (duloxetine), and predicted occupancies correlated moderately with PET data (*r* = 0.76, *p* = 0.028, R² = 0.58).

**Conclusions:**

These findings suggest that variability in NET engagement across psychotropic medications may not be fully captured by conventional class-based classifications. The proposed framework offers a mechanism-informed approach to comparative pharmacological analysis and introduces a conceptual noradrenergic activity index. This approach may be useful for hypothesis generation in future studies integrating pharmacokinetic-pharmacodynamic modeling with in vivo imaging and clinical outcomes.

## Highlights

- High occupancy is driven by either strong affinity or a combination of moderate affinity and low protein binding.
- Duloxetine shows greater NET occupancy than venlafaxine even at higher doses; metabolites (e.g. desvenlafaxine, norquetiapine, hydroxybupropion) contribute meaningfully.
- Low NET occupancy (e.g. paroxetine, ziprasidone) can still produce functional inhibition, reflecting non-linear pharmacodynamics.
- High-potency desipramine-equivalent dosing enables estimation of cumulative noradrenergic activity.
- Predicted NET occupancy correlated significantly with PET-derived estimates; however, plasma-based models do not fully capture central nervous system distribution (e.g. blood-brain barrier penetration), underscoring the need for validation in larger PET studies.

## Introduction

The concept of Different Dosage, Different Pharmacology (DDDP), introduced by the Neuroscience-based Nomenclature (NbN) task force, emphasizes that psychotropic medications may exert distinct clinical effects across dose ranges by engaging different neurobiological targets (Zemach et al., 2026). Within this framework, norepinephrine transporter (NET) inhibition may emerge progressively with dose escalation, reflecting a shift in pharmacological mechanism at higher exposures. Despite the central role of NET in psychopharmacology, current prescribing or deprescribing practices remain largely categorical, guided by drug class designations and regulatory labels rather than quantitative assessments of target engagement. Medications are often classified as “NET inhibitors” or “serotonin norepinephrine reuptake inhibitors (SNRIs)” based on in vitro binding data alone, without consideration of the complex interplay among binding affinity, plasma drug concentrations, and protein binding that may determine actual in vivo receptor occupancy (Owens et al., 1997).

Positron emission tomography (PET) imaging has emerged as the gold standard for measuring NET occupancy in vivo, providing direct quantification of target engagement in the human brain (Arakawa et al., 2019; Ding et al., 2014; Moriguchi et al., 2025; Nogami et al., 2013; Nyberg et al., 2013; Scherf-Clavel et al., 2020; Sekine et al., 2010; Takano et al., 2014). However, PET studies are resource-intensive, limited to specialized research centers, and available for only a small subset of psychotropic medications. Consequently, clinicians lack practical tools to compare noradrenergic potency across drugs or to predict the degree of NET engagement at clinically relevant doses. Pharmacometric modeling offers a complementary approach by integrating in vitro binding affinity data with pharmacokinetic parameters to estimate receptor occupancy across a broad range of medications (Roth et al., 2000).

Previous occupancy modeling efforts have primarily focused on dopamine and serotonin transporters (Nyberg et al., 2013), with limited application to NET. This study does not generate new experimental pharmacokinetic, pharmacodynamic, or PET imaging data; rather, it integrates previously published receptor affinity, protein binding, plasma exposure, and occupancy data into a unified pharmacometric framework to estimate NET engagement across psychotropic medications. The objectives of this study were to: (1) develop a pharmacometric framework for estimating NET occupancy across a comprehensive panel of psychotropic medications; (2) incorporate protein-binding corrections and active metabolite contributions into occupancy estimates; (3) derive target occupancy-based dose equivalents to enable standardized cross-drug comparisons; and (4) evaluate model predictions against published PET occupancy data.

We hypothesized that a mechanism-based occupancy model would reveal substantial heterogeneity in noradrenergic target engagement among medications within the same therapeutic class and provide a quantitative foundation for neuroscience-based treatment selection and prediction of noradrenergic adverse-event burden using a conceptual noradrenergic activity index (NAI).

## Methods

### Study design

This study employed a quantitative modeling approach to estimate NET occupancy across psychotropic medications. The framework integrates in vitro binding affinity (Ki) with free therapeutic plasma concentrations within the treatment-acceptable dosage range to predict in vivo NET occupancy (Sekine et al., 2010).

This framework assumes proportionality between plasma free concentration and target-site exposure, which may not fully account for central nervous system pharmacokinetics, including blood-brain barrier transport and tissue distribution.

### Data sources

Ki values for NET binding were obtained from the National Institute of Mental Health Psychoactive Drug Screening Program Ki database (https://pdsp.unc.edu), a publicly available repository of published and internally derived radioligand binding affinity data maintained by the laboratory of Bryan L. Roth at the University of North Carolina at Chapel Hill.

To identify psychotropic medications with potentially meaningful NET binding, an initial screening threshold of Ki ⩽3000 nM was applied. This threshold was selected to exclude compounds with negligible affinity while retaining agents with potential pharmacological relevance across therapeutic concentration ranges.

Only human-derived NET assays were incorporated, including studies performed in human cortical tissue and cloned human receptors. Data derived from non-human species were excluded. When multiple eligible Ki values were available for a given drug-target pair, a geometric mean was calculated to obtain a representative affinity estimate, which is provided in a Supplemental Table along with the individual contributing Ki values and their ranges. For compounds with only a single reported eligible Ki value, a geometric mean could not be calculated, and the reported value was retained.

The Psychoactive Drug Screening Program database contains both literature-derived and in-house affinity measurements. No preferential weighting was applied to either source, and all eligible human-derived observations meeting the inclusion criteria were considered. Only two in-house Ki values met the eligibility criteria and were incorporated into the final dataset. Active metabolites with known NET affinity were included when relevant.

Reported plasma concentration ranges were obtained from published pharmacokinetic drug-monitoring studies and reference databases, including David Knox et al. (2024) and the National Library of Medicine (2025), and were used in subsequent occupancy analyses. All sources provided in Table 1. These ranges were selected to capture a broad spectrum of clinically observed plasma exposures and estimate minimum, mean, and maximum NET occupancy, rather than to define validated therapeutic concentration ranges associated with efficacy or tolerability.

**Table 1.** Pharmacometric parameters of psychotropic drugs and metabolites.

Psychotropic drug or metabolite	Dose range (mg)	Geometric mean of NET binding affinity, Ki (nM)	Reported plasma concentrations range (nM)	Protein binding (% bound, f_u_)
Desipramine	100–300	2.47	375–1126	90, 0.1
Nortriptyline	25–150	4.6	190–570	93, 0.07
Atomoxetine	40–100	5	53–315	98, 0.02
Duloxetine	30–120	6.33	100–403	97, 0.03
Maprotiline	75–225	11.1	360–1442	88, 0.12
Reboxetine	8–12	13.2	262–357	97, 0.03
Doxepin	75–300	29.5	537–895	80, 0.2
Milnacipran	100–200	66.9	406–1218	13, 0.87
Ziprasidone	40–200	44	121–315	99, 0.01
Clomipramine	25–300	45.2	700–1591	97, 0.03
Imipramine	100–300	65.6	71–250	90, 0.1
Paroxetine	20–60	95.2	61–182	95, 0.05
Norquetiapine	22–712^a^	58	169–1693	83, 0.17
Amitriptyline	50–150	46.5	270–631	95, 0.05
Mianserin	30–90	71	189–340	90, 0.1
Dextromethorphan	45–90	240	180–295	60, 0.4
Methylphenidate	10–90	303	26–43	15, 0.85
Norsertraline	48–380^b^	908	171–1143	98, 0.02
Norclozapine	240–480^c^	494	1119–1918	95, 0.05
Fluoxetine	20–80	794	294–970	94, 0.06
Desvenlafaxine	41–206^d^	2753	72–1910	30, 0.7
Sertraline	50–400	808	98–653	98, 0.02
Quetiapine	25–800	1000	261–2610	83, 0.17
Bupropion	150–300	1539	42–420	84, 0.16
Hydroxybupropion	37.5–75^e^	940	3324–11,750	75, 0.25
Venlafaxine	75–375	1821	72–1911	27, 0.73

Source: Alkalay et al., 1980; Arvidsson et al., 2022; Axsome Therapeutics Inc, 2025; Breivik et al., 2024; Cellini et al., 2022; Chermá et al., 2008; Fleishaker et al., 2000; Hansen et al., 2017; Knadler et al., 2011; Kragh-Sørensen et al., 1973; Laib et al., 2014; Li et al., 2012; Mach et al., 2024; Maguire et al., 1982; Nelson et al., 1982; National Library of Medicine (n.d.) DailyMed. Available at: https://dailymed.nlm.nih.gov/dailymed/ (accessed 28 May 2026); Ostad Haji et al., 2013; Patel et al., 1996; Rigal et al., 1987; Sugimoto et al., 2021; Tomita et al., 2014; Vandel et al., 1978.

NET: Norepinephrine transporter.

a 89% quetiapine metabolized by CYP3A4.

b 95% sertraline metabolized by CYP2B6, CYP3A4, and others.

c 80% clozapine metabolized by CYP1A2 and CYP3A4 to norclozapine.

d 55% venlafaxine is converted by CYP2D6.

e 25% bupropion is converted by CYP2B6.

## Data analysis

NET occupancy was predicted to use a standard receptor binding model based on the standard concentration-to-affinity ratio:

$$Occupancy\left(\%\right)=\frac{C_{free}}{C_{free}+K_{i}}\times 100$$

where *C_free_* represents the unbound (free) plasma concentration of the drug, and *Ki* is the NET binding affinity constant. This equation assumes that free plasma concentrations are in equilibrium with brain concentrations at the NET binding site, a well-established assumption for centrally acting psychotropic medications that readily cross the blood-brain barrier (Hammarlund-Udenaes et al., 2008). Total plasma concentrations were corrected for protein binding to derive free drug concentrations: *C_free_* = *C_total_* *×* *fu.* Where *fu* is the fraction of drug unbound in plasma. This correction is critical because only the unbound fraction is pharmacologically active and available to distribute and bind to NET in the central nervous system.

For each compound, minimum, mean, and maximum occupancy values were calculated using the lower and upper bounds of the reported plasma concentration ranges.

## Target occupancy-based dose estimates (ED50)

To enable standardized comparison of noradrenergic potency across medications, the doses required to achieve a predefined level of NET occupancy (50%) (ED50) were calculated for each compound.

For each drug, the mean therapeutic dose was defined as the midpoint of the reported dose range and paired with the corresponding mean corrected NET occupancy.

Dose estimates for target occupancy were derived using a logit transformation of the occupancy function, based on the relationship:

$$logit\left(Occupancy\right)=log(\frac{Occupancy}{1-Occupancy})$$

These thresholds were selected based on PET literature suggesting that 50% occupancy represents a clinically meaningful level of target engagement (Nogami et al., 2013; Takano et al., 2014).

Assuming proportionality between dose and free drug concentration within the therapeutic range, target doses were calculated as:

$$ED_{t}=Dose_{mean}\times \frac{\left(\frac{Occ_{t}}{1-Occ_{t}}\right)}{\left(\frac{Occ_{mean}}{1-Occ_{mean}}\right)}$$

where *Occ_t_* represents the target occupancy (0.5), and *Occ_mean_* represents the mean protein-binding-corrected occupancy for each drug, as obtained from the logit transformation.

Intuitively, this approach scales dose according to how far a drug’s typical occupancy is from the 50% target, assuming roughly linear pharmacokinetics in the therapeutic range.

## Desipramine equivalence, relative potency and NAI

Desipramine was selected as the reference standard due to its high potency. For each medication, desipramine equivalence and relative potency were calculated as follows:

$$\begin{array}{l} Desipramine\;Equivalent\;Dose\;Ratio\\ \;=\;Drug\;ED50\;/\;Desipramine\;ED50 \end{array}$$

Indicates how many times more of the drug is needed compared to desipramine.

RelativePotency=DesipramineED50/DrugED50

Indicates the potency relative to desipramine.

The NAI uses additive scores to facilitate theoretical comparisons between psychotropic medications. This method evaluates a conceptual framework for noradrenergic activity and may enable ranking the cumulative activity of medications with NET-inhibiting effects for theoretical comparison.

NAI scores were assigned based on desipramine’s relative potency. The following thresholds are heuristic, chosen to approximate orders of magnitude in relative potency, not empirically derived clinical:

**Score 3 (High Index)**: Relative potency ⩾ 0.5
• Medications in this category have NET inhibition potency at least half that of desipramine
• May be associated with substantial noradrenergic effects at therapeutic doses
**Score 2 (Moderate Index)**: Relative potency 0.1 to <0.5
• Medications with moderate NET inhibition potency
• May produce clinically relevant noradrenergic effects
**Score 1 (Low Index)**: Relative potency 0.01 to <0.1
• Medications with weak NET inhibition potency
• May produce noradrenergic effects, particularly at higher doses or in sensitive individuals
**Score 0 (Minimal Index)**: Relative potency < 0.01
• Medications with negligible NET inhibition potency
• Unlikely to contribute meaningfully to noradrenergic activity at therapeutic doses

## Correlation analysis

For validation, predicted NET occupancy was compared with published PET-derived occupancy estimates. The association between predicted and PET-derived values was assessed using Pearson’s correlation coefficient (r), and the proportion of variance explained (R²), and the root mean square error were quantified. Linear regression was performed to assess agreement and detect systematic bias.

## Sensitivity analyses

Sensitivity analyses were conducted to assess the impact of uncertainty in key model parameters, including Ki values (±50% variation), protein binding fractions (±10% variation), and plasma concentration ranges (interquartile range). These analyses evaluated the robustness of occupancy estimates and dose equivalents to parameter uncertainty.

## Statistical analysis

All analyses were conducted using R version 4.5.2 and STATA BE version 19. A two-tailed *p*-value < 0.05 was considered statistically significant. Figures were generated using ggplot2 and related visualization packages.

## Ethics committee approval

Ethics approval was not required for this study, as it was based exclusively on data derived from published literature and publicly available databases.

## Results

Twenty-six psychotropic drugs and metabolites met the initial screening threshold (Ki ⩽ 3000 nM at human NET) and were carried forward into the occupancy analysis (Table 1). The highest NET affinity (lowest Ki values) was observed in the tricyclic antidepressants, desipramine (2.47 nM) and nortriptyline (4.6 nM). These were followed by atomoxetine (5.36 nM) and duloxetine (6.33 nM). Agents such as maprotiline, reboxetine, milnacipran, and ziprasidone showed intermediate-to-high affinities, with Ki values ranging from ≈11 to 44 nM. Paroxetine (95.2 nM) and mianserin (71 nM) exhibited intermediate-low affinities at the NET.

Selective serotonin reuptake inhibitors (SSRIs) like fluoxetine (794 nM) and sertraline (808 nM) demonstrated lower affinities, while bupropion (1539 nM) and the SNRI, venlafaxine (1821 nM), showed the lowest NET affinities.

Several major active metabolites exhibited notable NET affinity. These include norquetiapine (Ki = 58 nM), norclozapine (the metabolite of clozapine, Ki = 494 nM), and hydroxybupropion (the metabolite of bupropion, Ki = 940 nM).

Hydroxybupropion reported plasma concentrations (3324–11750 nM) are significantly higher than those of its parent drug, bupropion. This suggests potential functional binding with NET despite its relatively weak affinity.

Protein binding ranged from 13% (milnacipran) to 99% (ziprasidone). The fraction unbound (fu) consequently varied from 0.01 (ziprasidone) to 0.87 (milnacipran), reflecting substantial differences in free drug availability at the target site.

The minimum, mean, and maximum NET occupancy for each drug within its reported plasma concentration range is shown in Figure 1. After correcting for plasma protein binding, predicted NET occupancy was categorized as very high (⩾90%), high (70%–90%), moderate (50%–70%), Moderate-low (30%–50%), or low (⩽30%).

**Figure 1.**
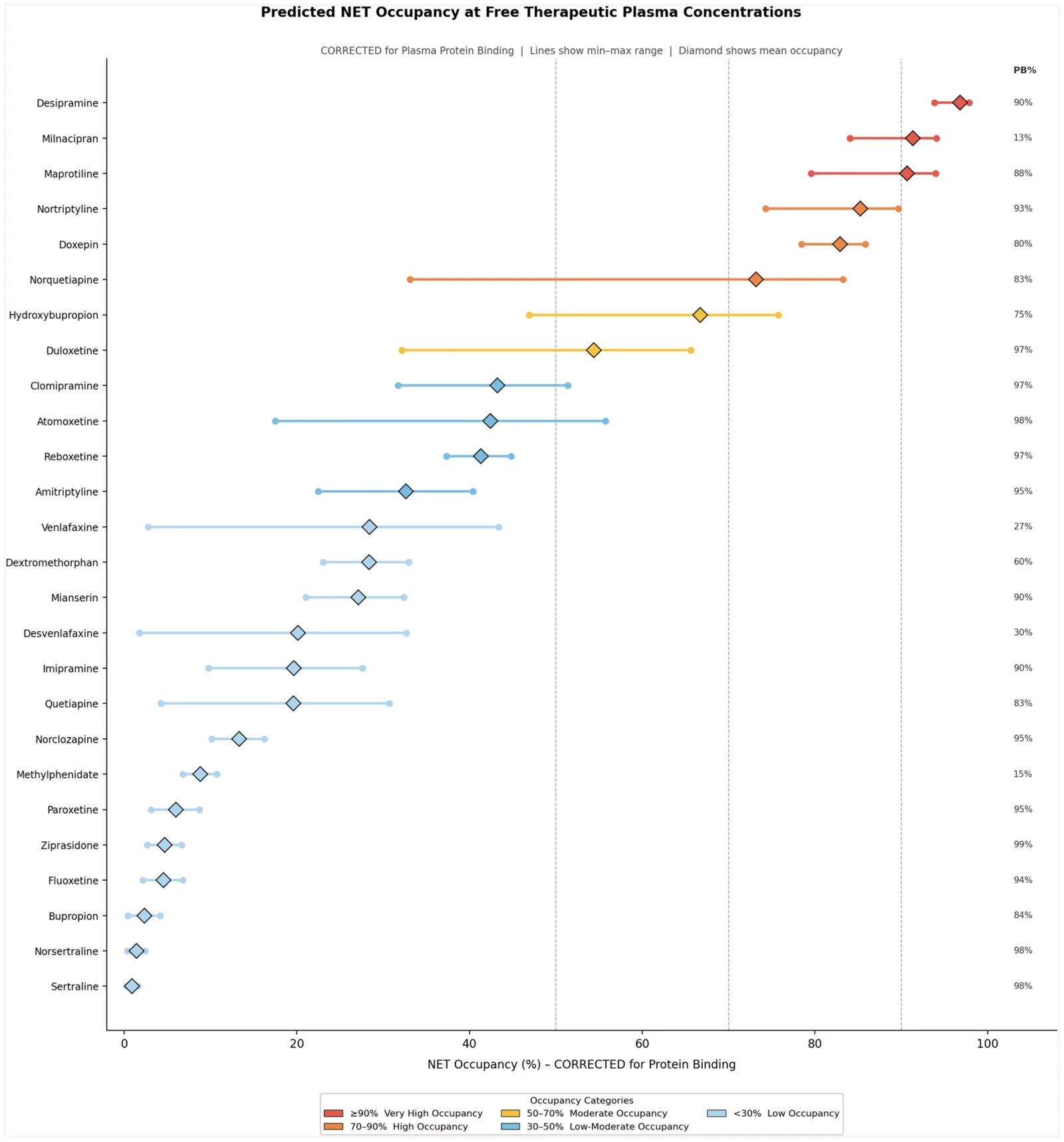
Predicted NET occupancy at free therapeutic plasma concentrations. *Note.* NET: Norepinephrine transporter.

Very high occupancy (⩾90%) drugs included desipramine (90% protein-bound), milnacipran (13% protein-bound), and maprotiline (88% protein bound). Notably, these agents represent opposite extremes of protein binding, with milnacipran reaching very high occupancy due to its exposure in the free fraction.

High occupancy (70%–90%) drugs included nortriptyline (93% protein-bound) and doxepin (80% protein-bound). Despite nortriptyline’s high protein binding, its very low Ki values were sufficient to maintain free-concentration-driven NET binding across the therapeutic range. The min-max ranges for these agents were relatively narrow, reflecting modest variability in therapeutic plasma concentrations. On the other hand, norquetiapine has a high occupancy of ≈75% but a wide range due to variable doses and extremely broad plasma concentrations (339–84,626 nM).

Hydroxybupropion and duloxetine achieved moderate predicted NET occupancy (50%–70%). Hydroxybupropion, the major active metabolite of bupropion, demonstrated a mean occupancy of approximately 68%, substantially exceeding that of the parent compound (2%). Despite its relatively weak NET affinity (Ki = 1700 nM) and moderate protein binding (~75%), hydroxybupropion achieved clinically meaningful occupancy owing to markedly higher steady-state plasma concentrations than bupropion. In contrast, bupropion itself demonstrated negligible NET occupancy, consistent with its weak NET affinity, high protein binding.

Moderate-low (30%–50%) occupancy included clomipramine (97% protein-bound), atomoxetine (98% protein-bound), which showed 42% occupancy (range 18%–56%), similar to reboxetine at 41% (range 37%–45%). The wide range of atomoxetine reflects both the breadth of its therapeutic plasma concentration spectrum and the substantial attenuation imposed by its very high protein binding (f_u_ = 0.02), as well as the influence of CYP2D6 metabolizer polymorphism status. Whereas reboxetine is metabolized by CYP3A4 with fewer polymorphisms.

Low occupancy (<30%) was observed in most agents after protein-binding correction. Venlafaxine achieved approximately 28% of the mean NET occupancy at standard therapeutic doses, despite its classification as an SNRI. This finding reflects venlafaxine’s substantially weaker NET affinity (Ki = 1821 nM) compared with duloxetine (Ki = 6.33 nM). The active metabolite O-desmethylvenlafaxine (desvenlafaxine) contributed an additional 20% mean occupancy. Notably, desvenlafaxine exhibited a range spanning 2% to 33%, reflecting the broad therapeutic concentration window reported for this compound. This may indicate that higher doses of venlafaxine’s NET inhibition are partly due to the accumulation of its metabolite.

Ziprasidone (99% protein-bound) showed a very low mean occupancy of approximately 5%, partly attributable to its near-complete plasma protein binding (fu = 0.01). Similarly, norclozapine (95% protein-bound) demonstrated low mean occupancy of approximately 13 (range 10%–16%).

SSRIs generally demonstrated minimal NET occupancy (<10%). Among the SSRIs, paroxetine achieved approximately 9% occupancy at high therapeutic concentrations. Fluoxetine, sertraline, and its major metabolite, norsertraline, all demonstrated mean occupancies below 10%. Norfluoxetine did not meet the criteria of Ki ⩽ 3000nM.

Using protein-binding-corrected NET occupancy predictions, dose-response relationships were standardized by calculating the doses required to achieve 50% (ED50) NET occupancy across psychotropic medications (Figure 2(a) and (b)). Predicted ED50 values varied substantially across compounds. Among antidepressants, desipramine demonstrated the highest potency (ED50 = 6.6 mg), serving as the reference compound. Milnacipran and reboxetine (ED50 = 14 mg) were comparably potent, followed by nortriptyline and maprotiline (ED50 = 15 mg). Duloxetine required a lower dose to achieve 50% occupancy (ED50 = 63 mg) than desvenlafaxine and venlafaxine. NET occupancy based on desvenlafaxine alone would require doses exceeding 400 mg/day, which corresponds to approximately 620 mg/day of venlafaxine, assuming 55% metabolic conversion. However, this estimate should be interpreted cautiously, as both venlafaxine and desvenlafaxine are pharmacologically active and contribute to NET inhibition. Consequently, the overall noradrenergic effect of venlafaxine treatment reflects the combined occupancy of the parent compound and its active metabolite, rather than the occupancy attributable to desvenlafaxine alone. Several metabolites demonstrated greater predicted NET engagement than their parent compounds: hydroxybupropion: ED50 = 28 mg versus bupropion 9367 mg, and norquetiapine, ED50 = 134 mg versus quetiapine 1690 mg. Marked heterogeneity was observed both within and between drug classes. This supports the DDDP/NbN concept that pharmacology may be more informative than traditional drug labels.

**Figure 2.**
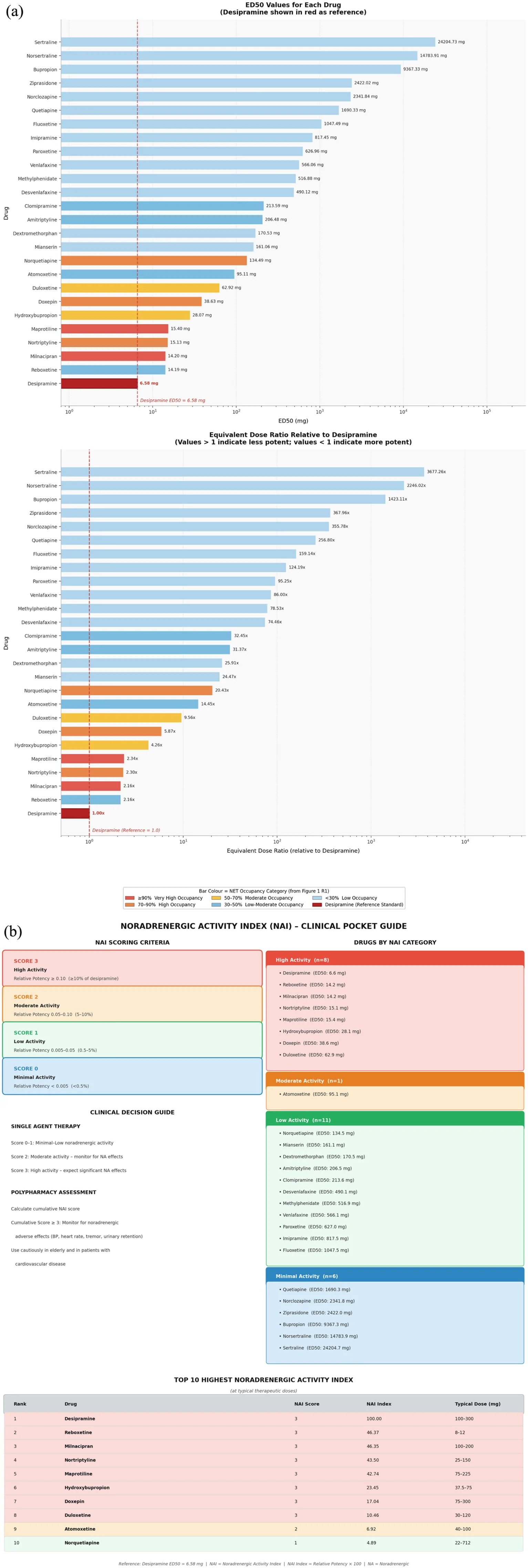
(a) ED50 and desipramine dose equivalence. (b) NAI conceptual framework. *Note.* NAI: Noradrenergic Activity Index.

PET-derived occupancy data were available for eight compounds and used for exploratory validation (Figure 3(a)). Predicted and PET-derived occupancies were moderately correlated (*r* = 0.76, *p* = 0.028, R² = 0.58), explaining approximately 58% of variance. Overall, corrected occupancy estimates were generally consistent with PET findings, although notable drug-specific discrepancies were observed. The largest overestimations occurred for milnacipran (89.3% vs. 50.0%; Δ = +39.3% points), nortriptyline (89.7% vs. 70.0%; Δ = +19.7% points), and norquetiapine (44.3% vs. 30.0%; Δ = +14.3% points). In contrast, the model underestimated occupancy for quetiapine (10.9% vs. 35.0%; Δ = −24.1% points) and desvenlafaxine (27.9% vs. 37.0%; Δ = −9.1% points). Agreement was strongest for venlafaxine (49.2% vs. 50.0%; Δ = −0.8% points), duloxetine (48.0% vs. 50.0%; Δ = −2.0% points), and atomoxetine (86.8% vs. 80.0%; Δ = +6.8% points). Notably, milnacipran was identified as an outlier, exhibiting a large overestimation of 89.3% versus 50% in PET studies, likely attributable to its unusually low plasma protein binding (13%), which may cause the correction formula to overestimate occupancy possibly reflecting active transport or regional brain distribution differences not captured by the plasma concentration-based model. Removing milnacipran substantially increases the fit and hence validity of the model (Pearson *r* = 0.8933, *p* = 0.0067, R² = 0.7980) (Figure 3(b)). The coefficient of determination (R² = 0.7980) indicated that approximately 80% of the variability in PET-measured NET occupancy was explained by the model’s predicted occupancy values. See Supplemental Material for the raw results.

**Figure 3.**
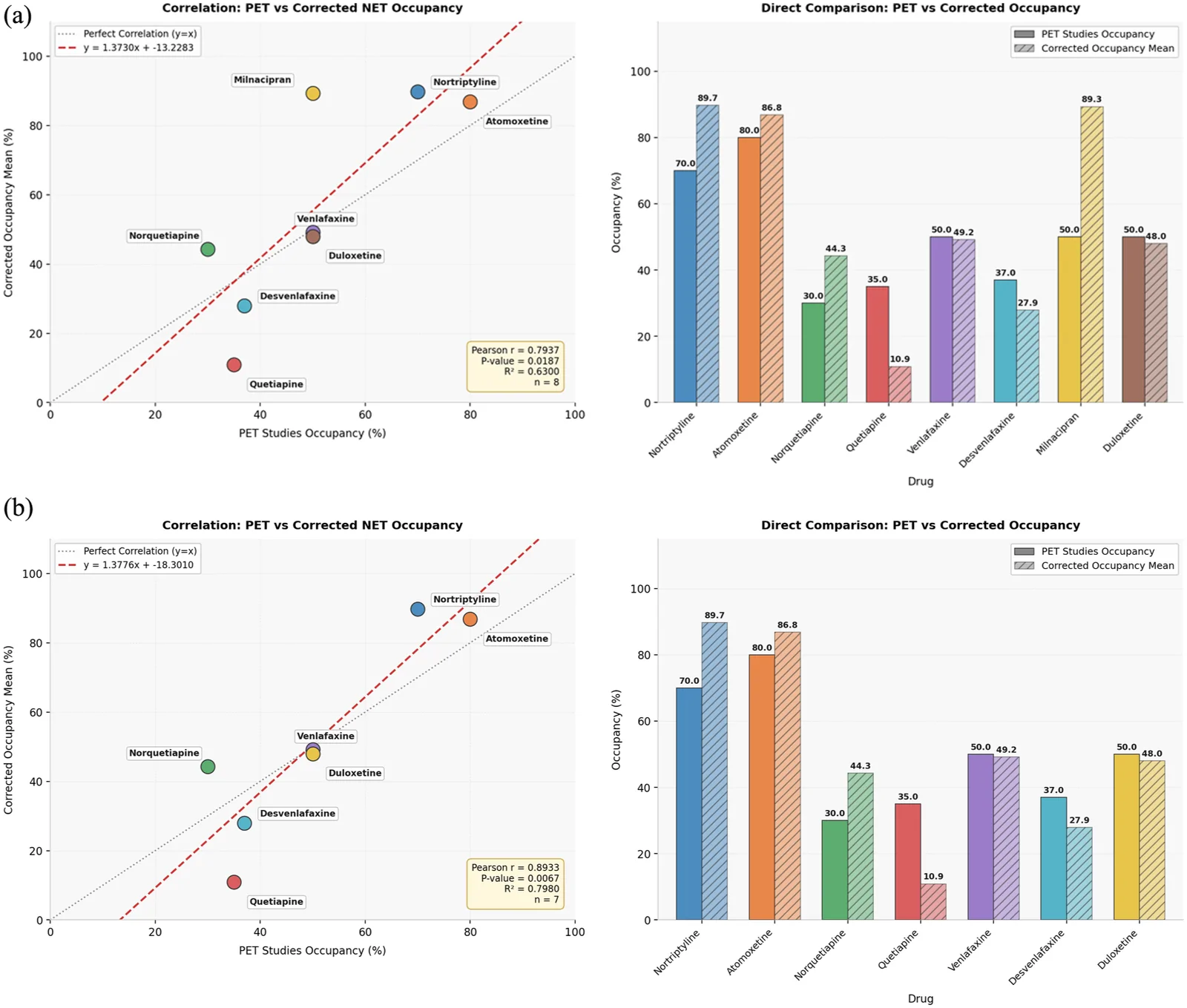
(a) Predicted NET occupancy compared with NET occupancy from PET studies (b) Predicted NET occupancy compared with NET occupancy from PET studies without milnacipran. *Note.* NET: Norepinephrine transporter; PET: Positron emission tomography.

The sensitivity analyses demonstrated that predicted NET occupancy is influenced primarily by uncertainty in binding affinity (Ki) and plasma exposure, whereas uncertainty in protein binding (fu) has a comparatively modest effect. Variations in Ki and plasma concentrations produced similar average changes in predicted occupancy (mean ΔOcc = 15.2% and 16.0%, respectively), while changes in fu resulted in substantially smaller effects (mean ΔOcc = 2.74%). Importantly, uncertainty in Ki values led to the highest frequency of occupancy-category transitions (65% of compounds), highlighting the critical importance of accurate affinity estimates for pharmacometric modeling. Drugs within the intermediate occupancy range (approximately 30%–70%) were most sensitive to parameter variation because they occupy the steepest portion of the occupancy curve, where small changes in exposure or affinity can produce substantial changes in predicted target engagement. In contrast, compounds with very high baseline occupancy (⩾90%) were relatively robust to parameter uncertainty due to saturation kinetics, whereby further increases in exposure produce only minimal changes in occupancy. The high frequency of category transitions observed in the Ki- and exposure-based sensitivity analyses underscores the importance of precise pharmacokinetic and pharmacodynamic characterization when interpreting comparative NET occupancy estimates. See Supplemental Material.

## Discussion

To our knowledge, this is the first study to propose a comprehensive pharmacometric framework to estimate NET occupancy across psychotropic medications by integrating transporter binding affinity, drug exposure, and protein binding. The findings suggest that NET occupancy varies substantially across agents and may not be reliably inferred from drug class alone. In addition, pharmacokinetic factors, particularly protein binding and active metabolites, appear to meaningfully influence target engagement. These results support the potential utility of target-occupancy-based dose estimates as a standardized, though theoretical, approach for comparing noradrenergic effects across medications.

Several agents, including desipramine and nortriptyline, were predicted to achieve relatively high NET occupancy due to their high binding affinity. In contrast, maprotiline and doxepin achieve this through a combination of moderate affinity and lower protein binding.

Within SNRIs, notable differences emerged. Duloxetine showed moderate-to-high predicted occupancy, whereas venlafaxine demonstrated relatively low occupancy at typical doses, likely reflecting its weak NET affinity. The active metabolite, desvenlafaxine, showed a wide occupancy range, consistent with evidence that clinically meaningful NET engagement with venlafaxine may occur primarily at higher doses (>150 mg/day) (Arakawa et al., 2019; Scherf-Clavel et al., 2020). These findings align with meta-analytic data suggesting only modest differences between venlafaxine and SSRIs in clinical outcomes (de Silva and Hanwella, 2012; Nemeroff et al., 2008) and may indicate that noradrenergic effects are dose-dependent and variable across agents.

Taken together, these findings suggest that the clinical distinction between SNRIs and SSRIs may be less pharmacodynamically meaningful for venlafaxine at typical doses than for duloxetine or milnacipran. Meta-analysis indicates that duloxetine does not differ from venlafaxine; however, dose plays a central role in such a comparison (Schueler et al., 2011). Future clinical studies could explore whether duloxetine is comparable to high-dose venlafaxine.

Atomoxetine demonstrated a wide range of predicted occupancy, potentially reflecting both high protein binding and known pharmacogenetic variability in CYP2D6 metabolism (Smith et al., 2023; Yu et al., 2016). This variability may contribute to interindividual differences in clinical response and tolerability. For example, somnolence is dose-dependent and influenced by individual variability in plasma drug concentrations and metabolism (Cheng et al., 2024).

The contribution of active metabolites was evident for several compounds, including norquetiapine and hydroxybupropion, supporting the importance of incorporating metabolite exposure into pharmacodynamic models (Yatham et al., 2018). Hydroxybupropion’s mean occupancy of approximately 65%, substantially exceeding that of its parent compound bupropion (<5%), illustrates a broader pharmacological principle: that active metabolites accumulating to high steady-state concentrations may be the principal mediators of its noradrenergic effects, even when the parent compound itself shows negligible transporter engagement. Additionally, this finding may have implications for CYP2B6-mediated interactions (the primary enzyme responsible for hydroxybupropion formation, Knox et al., 2024). Factors that alter hydroxybupropion formation or clearance, including CYP2B6 genetic polymorphisms and drug inhibitors/inducers, may substantially affect noradrenergic target engagement and clinical outcomes. This finding also suggests that therapeutic drug monitoring of hydroxybupropion, rather than bupropion alone, may be more informative for optimizing treatment.

A discrepancy was observed between the predicted NET occupancy (1%–10%) and the ex vivo inhibition of norepinephrine uptake by paroxetine (27%–43% inhibition) (Gilmor et al., 2002). This may reflect the non-linear relationship between transporter occupancy and functional inhibition, whereby relatively low occupancy can still produce measurable pharmacological effects. Consequently, occupancy estimates should not be interpreted as direct measures of clinical efficacy or adverse-effect liability. Rather, the present framework is intended primarily as a mechanistic exposure index and comparative pharmacological descriptor of predicted noradrenergic target engagement. Its purpose is to facilitate quantitative comparison among medications, doses, and active metabolites using a common biological metric.

Notably, the atypical antipsychotic ziprasidone has a high affinity for NET (Ki = 44 nM); however, its high protein binding results in only 5% predicted occupancy. Given its role in augmentation strategies for depression, the influence of NET might be more significant, though ziprasidone also acts on a wide range of receptors (Ionescu et al., 2016, Stahl and Shayegan, 2003). Fluoxetine and norsertraline, the active metabolite of sertraline, both demonstrated a predicted mean NET occupancy similar to paroxetine after protein-binding adjustments. Both may have noradrenergic effects at high plasma concentrations. However, a review of 522 trials with 116,477 participants found that fluoxetine had a lower response rate than paroxetine, while sertraline’s efficacy was comparable to that of paroxetine and other SSRIs (Cipriani et al., 2018).

An acceptable threshold of approximately 50% NET occupancy has been proposed as a marker of clinically relevant noradrenergic effects, although this remains somewhat arbitrary and likely drug-dependent, with effects seen at 30% occupancy (Takano et al., 2014, Moriguchi et al., 2025).

Within this context, target-based dose metrics (e.g. ED50) may offer a more clinically interpretable framework for comparing noradrenergic potency across agents. Expressing dose equivalence relative to desipramine provides a conceptual approach to estimating “noradrenergic activity index,” which may be relevant in polypharmacy settings. However, this framework should be considered preliminary and requires validation in prospective clinical PET studies.

Combining agents with low individual NET occupancy could still result in minimal cumulative noradrenergic effects, especially if their effective doses (ED50) exceed clinically achievable levels. For example, 1 mg of desipramine is estimated to be equivalent to 10 mg of duloxetine and 20 mg of norquetiapine.

As a theoretical illustration, a patient taking 600 mg of quetiapine (89% of which is converted to norquetiapine) and 120 mg of duloxetine, whose potency ratios to desipramine are 20:1 and 10:1, respectively, may have an estimated combined noradrenergic equivalent of approximately 39 mg of desipramine. This corresponds to an exposure approximately six-fold higher than the estimated desipramine ED50 for NET occupancy (6.6 mg). According to the conceptual framework guide, the NAI score for these two drugs is three, indicating a high level of noradrenergic activity (Figure 2(b)).

This should be interpreted as hypothesis-generating pending prospective validation. With further empirical validation, this approach may facilitate the development of a standardized noradrenergic burden or efficacy scale, enabling clinicians to better balance therapeutic efficacy against tolerability and cardiovascular risk in multi-drug regimens. Similarly, recent recommendations from the American Society of Clinical Psychopharmacology (Goldberg et al., 2026b) on psychotropic deprescribing emphasize the importance of recognizing pharmacodynamically redundant medications and minimizing unnecessary mechanistic overlap. Within this context, further validation of desipramine equivalence and NAI may provide a framework for predicting NET inhibition redundancy across psychotropic regimens.

## Correlation with PET derived occupancy and limitations

Comparison of predicted NET occupancy with PET-derived estimates showed a statistically significant positive correlation (*r* = 0.76, *p* = 0.028), explaining approximately 58% of the variance (R² = 0.58), despite the limited sample size (*n* = 8). The regression model indicated a systematic tendency for the pharmacometric approach to overestimate occupancy (Predicted Occupancy = 1.37 × PET Occupancy−13.2), suggesting that plasma-based estimates may not fully account for physiological constraints, such as blood-brain barrier penetration and tissue distribution. Three of the eight evaluated drugs demonstrated good-to-excellent agreement with PET data, with absolute prediction errors below 10%, and particularly strong concordance was observed for atomoxetine (6.8%), duloxetine (2.0%), and venlafaxine (0.8%). In contrast, milnacipran emerged as a notable outlier, with substantial overestimation of ~38.3% greater predicted occupancy, likely attributable to its unusually low plasma protein binding (13%), which may lead to disproportionate inflation of free drug-based occupancy estimates in the model. When milnacipran was removed, the correlation was notably stronger (*r* = 0.8933, *p* = 0.0067, R² = 0.7980). Overall, these findings support the directional validity of the framework while emphasizing that it should be interpreted as a semi-quantitative tool for relative comparison rather than precise prediction of in vivo central occupancy, pending validation in larger datasets with direct PET measurements.

A notable limitation of the present framework is that the relationship between NET occupancy and clinical outcomes remains incompletely established. Unlike dopamine D2 receptor occupancy, for which clinically relevant thresholds have been derived through extensive PET-clinical correlation studies, no analogous occupancy threshold has been validated for NET. Consequently, the occupancy categories used in this study (“low,” “moderate,” “ low-moderate,” “high,” and “very high”) should be regarded as descriptive and relative, based on the distribution of predicted occupancy values across agents rather than on established clinical benchmarks. Similarly, the 50% occupancy threshold used to calculate ED50 values was adopted from convention in the PET literature and prior occupancy studies, where it has been used as an operational marker of meaningful target engagement, rather than from direct evidence linking this threshold to clinical efficacy, tolerability, or other patient outcomes (Nogami et al., 2013; Takano et al., 2014). Accordingly, the framework estimates predicted NET occupancy derived from in vitro affinity, protein binding, and plasma exposure parameters and should be viewed as a pharmacokinetic-pharmacodynamic surrogate rather than a measure of clinical effect. Its primary utility lies in enabling quantitative cross-drug comparisons, ranking medications by predicted noradrenergic target engagement, and evaluating the contribution of active metabolites within a standardized framework. However, the model does not establish any causal or quantitative relationship between predicted occupancy and antidepressant response, speed of onset, symptom improvement, adverse effects, or other clinically meaningful outcomes. Determining whether higher NET occupancy translates into greater therapeutic benefit, differential effects across symptom domains, or improved outcomes in specific patient populations will require prospective studies integrating pharmacometric predictions, PET imaging, therapeutic drug monitoring, and clinical outcome measures. The present framework is intended to facilitate and inform such investigations rather than to resolve these questions.

We assumed a direct equilibrium relationship between unbound plasma concentration and target occupancy of the form $Occ=\frac{C_{f}}{C_{f}+K_{i}}\times 100$ , which is mathematically equivalent to standard Emax models used to describe NET occupancy for several antidepressants. However, some drugs in our panel have complex central nervous system pharmacokinetics and multi-target pharmacology, for which occupancy may not be fully determined by the instantaneous unbound plasma concentration and a single in vitro Ki. Thus, several additional limitations warrant consideration. First, the model does not account for adaptive neurobiological changes associated with chronic exposure, such as changes in transporter density or function, which may alter the relationship between occupancy and clinical effect over time. Second, NET occupancy captures only a single dimension of noradrenergic pharmacology; downstream processes, including postsynaptic receptor sensitivity, intracellular signaling, and presynaptic autoreceptor feedback, are not incorporated and may substantially influence both therapeutic and adverse outcomes. Third, the framework relies on integrating parameters derived from heterogeneous sources, including binding affinity (Ki) and therapeutic plasma concentration ranges obtained from studies conducted across different laboratories and methodologies. Variability in assay conditions, radioligand selection, and population characteristics may introduce systematic bias and inter-study heterogeneity, potentially affecting the precision and comparability of the estimated occupancy values.

The use of plasma-based exposure and protein-binding correction as a surrogate for target-site concentration does not account for several pharmacokinetic and pharmacodynamic processes that may influence the relationship between systemic exposure and in vivo NET occupancy. Temporal dissociation (hysteresis) may occur between plasma concentrations and central target engagement, particularly when equilibration between plasma and brain compartments is delayed. Also, drugs differ substantially in their brain-to-plasma partitioning, such that similar plasma concentrations may yield markedly different extracellular concentrations within the central nervous system (Gunn and Rabiner, 2017). In addition, the model assumes equilibrium between free plasma and brain extracellular drug concentrations and does not account for active efflux transporters such as P-glycoprotein (P-gp), breast cancer resistance protein, and other ATP-binding cassette transporters. As several psychotropic medications are known substrates of these transport systems, central nervous system free drug concentrations may differ substantially from free plasma concentrations despite similar systemic exposure. Differences in intracellular sequestration, tissue binding, and subcellular distribution may further alter the relationship between free plasma concentrations and transporter occupancy. Finally, synaptic exposure kinetics, including rates of brain entry, clearance, and transporter association and dissociation, may differ between compounds despite similar steady-state plasma exposure. Consequently, plasma-based occupancy models may overestimate central target engagement in compounds with significant transporter-mediated efflux or restricted brain penetration. These factors may explain discrepancies observed between predicted occupancy estimates and PET-measured in vivo occupancy.

## Positioning the framework within existing evidence

Despite these limitations, the present study provides a structured, mechanism-informed approach for comparing noradrenergic target engagement across psychotropic medications that could be developed and further clinically validated in prescribing or deprescribing rationale.

An important consideration is the nature of the contribution provided by this work. The framework is derived entirely from previously published binding affinity data, pharmacokinetic parameters, and a limited number of PET imaging studies; therefore, it does not provide new experimental or clinical evidence regarding NET engagement. Rather, its value lies in integrating these previously disparate data sources into a unified quantitative framework. Although clinicians and researchers may broadly recognize differences in the noradrenergic properties of psychotropic medications, such knowledge is often class-based, qualitative, and difficult to compare across compounds, dose ranges, metabolites, and drug classes. By translating affinity, protein binding, exposure, and occupancy data into common quantitative metrics, the framework enables systematic comparison of predicted NET engagement across medications that have rarely been evaluated within the same analytical context. Furthermore, the proposed desipramine-equivalent approach and NAI provide a reproducible methodology for quantifying and comparing potential noradrenergic contributions in both monotherapy and polypharmacy settings. Recent deprescribing recommendations may provide an additional rationale for developing such a framework. The recent American Society of Clinical Psychopharmacology (ASCP) Delphi consensus on psychotropic deprescribing identified antidepressants with duplicative mechanisms of action as candidates for discontinuation in major depressive disorder (Goldberg et al., 2026a, 2026b). However, current deprescribing recommendations largely rely on qualitative assessments of pharmacological similarity and lack quantitative measures of relative NET engagement among antidepressants. A standardized NET engagement index may therefore offer an objective method for comparing noradrenergic activity across medications, identifying potentially redundant noradrenergic mechanisms in antidepressant polypharmacy, and estimating the contribution of active metabolites such as hydroxybupropion and norquetiapine to overall NET inhibition.

Importantly, the framework generates testable predictions that can be prospectively evaluated using PET imaging, therapeutic drug monitoring, and clinical outcome studies. Thus, while the findings should be interpreted as a secondary pharmacometric synthesis rather than direct evidence of in vivo target engagement, the framework may serve as a useful tool for hypothesis generation, comparative psychopharmacology, study design, and the development of future translational models integrating pharmacokinetics, neuroimaging, and clinical outcomes.

## Conclusions

This study presents a pharmacometric integration of published in vitro affinity data, pharmacokinetic parameters, and available PET findings to estimate NET occupancy, desipramine dose equivalence, and a NAI across psychotropic medications. The findings highlight substantial variability in predicted noradrenergic engagement between agents and underscore the influence of pharmacokinetic factors and active metabolites on NET occupancy. As the framework is derived entirely from previously published pharmacological, pharmacokinetic, and imaging data, it does not provide new experimental or clinical evidence of NET engagement. Rather, it offers a quantitative synthesis intended to facilitate comparison of the potential noradrenergic effects of psychotropic medications. While the observed agreement with available PET studies supports the model’s plausibility, the framework should be regarded as hypothesis-generating and requires further validation through prospective PET imaging and clinical investigations. Consequently, it is not intended to guide individual clinical decision-making but may serve as a conceptual and research tool for understanding and studying noradrenergic pharmacology.

## Supplemental Material

sj-xlsx-1-jop-10.1177_02698811261470450 – Supplemental material for A pharmacometric framework for norepinephrine transporter occupancy and dose equivalence across psychotropic medications Supplemental material, sj-xlsx-1-jop-10.1177_02698811261470450 for A pharmacometric framework for norepinephrine transporter occupancy and dose equivalence across psychotropic medications by Mohammed Aboukaoud, Bosmat Hoch and Revital Amiaz in Journal of Psychopharmacology

sj-png-1-jop-10.1177_02698811261470450 – Supplemental material for A pharmacometric framework for norepinephrine transporter occupancy and dose equivalence across psychotropic medications Supplemental material, sj-png-1-jop-10.1177_02698811261470450 for A pharmacometric framework for norepinephrine transporter occupancy and dose equivalence across psychotropic medications by Mohammed Aboukaoud, Bosmat Hoch and Revital Amiaz in Journal of Psychopharmacology

sj-png-2-jop-10.1177_02698811261470450 – Supplemental material for A pharmacometric framework for norepinephrine transporter occupancy and dose equivalence across psychotropic medications Supplemental material, sj-png-2-jop-10.1177_02698811261470450 for A pharmacometric framework for norepinephrine transporter occupancy and dose equivalence across psychotropic medications by Mohammed Aboukaoud, Bosmat Hoch and Revital Amiaz in Journal of Psychopharmacology

## Data Availability

All data used in this study were obtained from publicly available sources. Binding-affinity (Ki) data are available through the National Institute of Mental Health Psychoactive Drug Screening Program Ki Database (https://pdsp.unc.edu). Clinical pharmacokinetic and PET data were extracted from published studies indexed in PubMed and Embase. The relevant primary sources are cited throughout the manuscript.
